# A nomogram with Nottingham prognostic index for predicting locoregional recurrence in breast cancer patients

**DOI:** 10.3389/fonc.2024.1398922

**Published:** 2024-09-16

**Authors:** Jianqing Zheng, Bingwei Zeng, Bifen Huang, Min Wu, Lihua Xiao, Jiancheng Li

**Affiliations:** ^1^ Department of Radiation Oncology, The Second Affiliated Hospital of Fujian Medical University, Quanzhou, Fujian, China; ^2^ Department of Pathology, The Second Affiliated Hospital of Fujian Medical University, Quanzhou, Fujian, China; ^3^ Department of Obstetrics and Gynecology, Quanzhou Medical College People’s Hospital Affiliated, Quanzhou, Fujian, China; ^4^ Department of Radiation Oncology, Clinical Oncology School of Fujian Medical University, Fujian Cancer Hospital, Fuzhou, China

**Keywords:** Nottingham prognostic index, primary breast cancer, locoregional recurrence, prediction model, prognostic analysis

## Abstract

**Background:**

The Nottingham prognostic index (NPI) has been shown to negatively impact survival in breast cancer (BC). However, its ability to predict the locoregional recurrence (LRR) of BC remains still unclear. This study aims to determine whether a higher NPI serves as a significant predictor of LRR in BC.

**Methods:**

In total, 238 patients with BC were included in this analysis, and relevant clinicopathological features were collected. Correlation analysis was performed between NPI scores and clinicopathological characteristics. The optimal nomogram model was determined by Akaike information criterion. The accuracy of the model’s predictions was evaluated using receiver operating characteristic curves (ROC curves), calibration curves and goodness of fit tests. The clinical application value was assessed through decision curve analysis.

**Results:**

Six significant variables were identified, including age, body mass index (BMI), TNM stage, NPI, vascular invasion, perineural invasion (*P*<0.05). Two prediction models, namely a TNM-stage-based model and an NPI-based model, were constructed. The area under the curve (AUC) for the TNM-stage- and NPI-based models were 0.843 (0.785,0.901) and 0.830 (0.766,0.893) in training set and 0.649 (0.520,0.778) and 0.728 (0.610,0.846) in validation set, respectively. Both models exhibited good calibration and goodness of fit. The F-measures were 0.761vs 0.756 and 0.556 vs 0.696, respectively. Clinical decision curve analysis showed that both models provided clinical benefits in evaluating risk judgments based on the nomogram model.

**Conclusions:**

a higher NPI is an independent risk factor for predicting LRR in BC. The nomogram model based on NPI demonstrates good discrimination and calibration, offering potential clinical benefits. Therefore, it merits widespread adoption and application.

## 1 Introduction

Breast cancer (BC) is one of the most prevalent and life-threatening malignant tumors affecting women worldwide, with high morbidity and mortality rates (1). According to the data from International Agency for Research on Cancer of World Health Organization, there were 2.26 million new cases of BC globally in 2020, accounting for 11.7% of new cancer cases. This means that BC has surpassed lung cancer for the first time and has become the most prevalent cancer in the world (2).

Radiotherapy is a crucial component of multimodal treatment for BC. It is used for early-stage, locally advanced and metastatic BC, especially in patients undergoing breast-conserving therapy and those with high-risk factors after modified radical mastectomy (3, 4). The main form of radiotherapy in comprehensive BC treatment is adjuvant radiotherapy (ART), although some cases with large tumors may require neoadjuvant radiotherapy. Numerous randomized controlled studies and systematic reviews have demonstrated that ART significantly reduces the risk of locoregional and distant BC failure and improves overall survival (3, 5, 6). However, despite advances in radiotherapy over the past two decades, locoregional recurrence (LRR) after radiotherapy remains the most significant treatment failure for BC (7, 8). LRR is a common failure mode in most BC cases and can be caused by radioresistance (9). Thus, identifying high-risk factors for cancer recurrence before radiotherapy can aid in determining the appropriate treatment approach and minimize adverse effects on patients. Nevertheless, this is challenging due to the relatively limited clinical indicators available for predicting cancer recurrence after radiotherapy (9, 10).

The Nottingham prognostic index (NPI) was developed by Haybittle in 1982 as a risk assessment tool for patients with BC. It calculates a score based on histopathological factors such as tumor size, lymph node status, and histological grade. The NPI remains one of the most important biological predictors for BC today (8, 11). By comprehensively considering tumor size, lymph node status, and histological grade, the NPI aids clinicians categorize patients into different prognostic groups, allowing for a more accurate prognosis prediction (12). Several studies have examined the relationship between overall survival and NPI, showing that higher NPI scores effectively predict worse long-term survival in breast cancer patients (13–15).

While higher NPI scores have been shown to negatively impact survival in BC, it is still unclear whether they can predict the risk of LRR. Therefore, the aim of this study was to determine whether higher NPI scores play a significant role in predicting LRR in BC by constructing two predictive models: a TNM staging-based prognostic model and an NPI-based prognostic model.

## 2 Materials and methods

### 2.1 Study subjects

**Figure 1:**
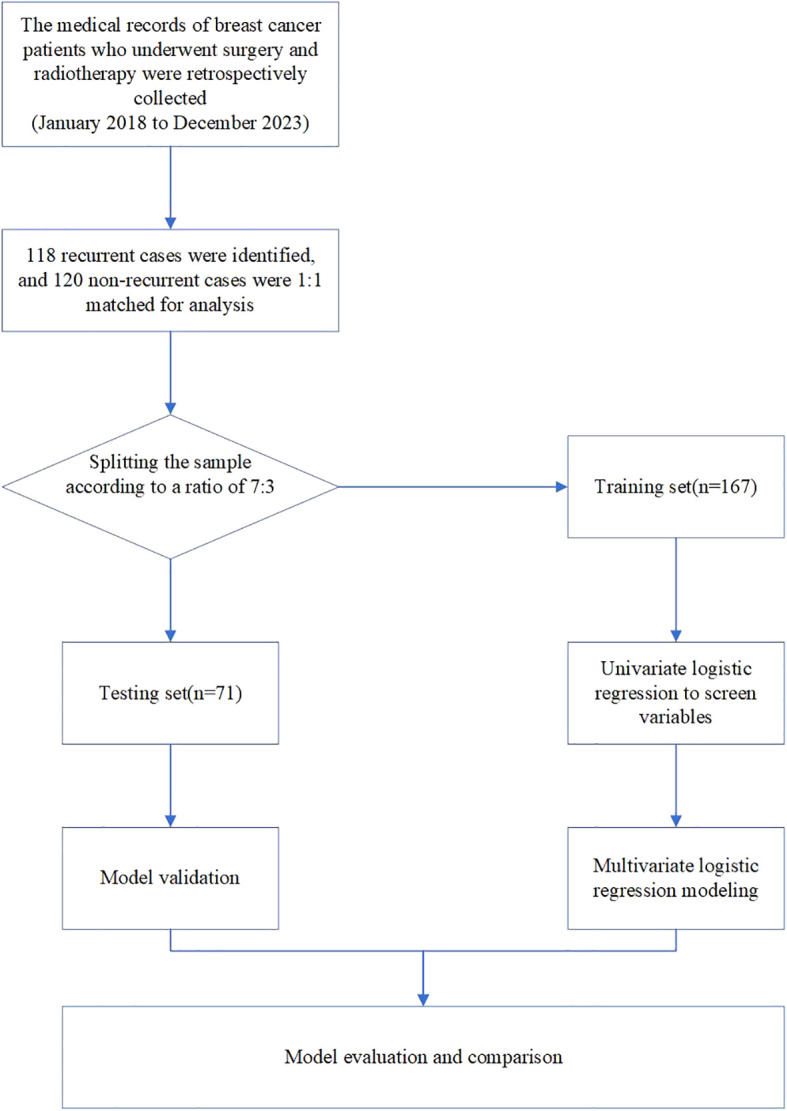
Flow chart of study design.

In total, 238 patients with BC from the Second Affiliated Hospital of Fujian Medical University were retrospectively reviewed between January 2018 and December 2023. To be included in the study, patients needed to meet the following criteria: (1) diagnosed with BC without distant metastatic disease (16), including all molecular subtypes. The pathological types of breast cancer included were invasive ductal carcinoma and invasive lobular carcinoma. (2) underwent standardized surgery, either breast-conserving surgery (BCS) or modified radical mastectomy, (3) received standard ART, either conventional fractionated radiotherapy or hypofractionated radiotherapy, (4) possessed detailed pathological and clinical information necessary for NPI calculation. The exclusion criteria were as follows: Those with (1) non-invasive ductal carcinoma, non-invasive lobular carcinoma, or other types of breast cancer. (2) severe underlying diseases. (3) concurrent tumor diseases or previous cancer diagnosis; and (4) patients who were untraceable during the follow-up period for evaluating tumor recurrence. To improve the prediction accuracy of the model, a 1:1 sample ratio was used for recurrent and non-recurrent cases. The flowchart of the study is shown in **Figure 1**. Finally, 118 patients with BC and locoregional recurrence were identified and matched with 120 patients with BC without locoregional recurrence during the same period. This retrospective study was approved by the Ethical Review Committee of the Second Affiliated Hospital of Fujian Medical University (2024–294).

### 2.2 Data collection, filtering and preparation

The basic information collected included age, body mass index (BMI), TNM staging, treatment status, tumor location. These data were obtained through the hospital’s electronic medical record system and pathological examination system. The important pathological features required for this study included tumor size (cm), lymph node metastasis status, tumor pathological grading, estrogen receptor status, and HER2 status. The clinical and pathological data of the included patients were collected by Bingwei Zeng and Jianqing Zheng.

### 2.3 Calculation principle and method of the NPI

Pathological examination was performed on excised tumor tissue, including tumor size and the metastasis status of axillary lymph nodes. Histological grading was conducted under the microscope using paraffin slide hematoxylin-eosin staining. NPI scores were calculated using the following formula: NPI = tumor size (cm) ×0.2+ lymph node metastasis (0-3 points) + histological grade (1-3 points). The points of lymph node involvement were recorded based on the number of positive lymph node metastasis (0 points for 0 positive lymph node, 1 point for 1-3 positive lymph nodes, 2 points for 4-9 positive lymph nodes and 3 points for more than >9 positive lymph nodes). Histological grading was scored according to tumor differentiation (1 point for well-differentiated tumor, 2 points for moderately differentiated tumor, and 3 points for poorly differentiated tumor). In clinical practice, three prognostic groups can be identified based on the total NPI score: NPI <3.4 indicates a good prognosis (NPI1), 3.4 to 5.4 indicates a moderate prognosis (NPI2), and >5.4 indicates a poor prognosis (NPI3).

### 2.4 Radiotherapy target volume and dose

For patients who underwent BCS in the early stages of breast cancer, the clinical target volume (CTV) of postoperative adjuvant radiotherapy included soft tissues of the whole breast down to deep fascia. The underlying muscle, ribcage, overlying skin, and excision scar were not included. The planning target volume (PTV) included the entire breast with 1-cm margins to encompass palpable breast tissue. Hypofractionated whole breast irradiation of 42.56 Gy/16 fractions was administered, and an additional boost irradiation of 10.64 Gy/4 fractions was administered when the surgical margin was ≤5 mm (17, 18).

For patients who underwent modified radical mastectomy, the CTV comprised the ipsilateral chest wall, mastectomy scar, and supra-/infraclavicular region. Each CTV was delineated according to the BC atlas for radiation therapy planning consensus definitions of the Radiation Therapy Oncology Group (RTOG) (19). The chest wall CTV was expanded 1 cm to become the chest wall PTV, except that the anterior, posterior and cranial borders, which remained unchanged. Whole breast irradiation of 45-50 Gy/25 fractions was administered, and an additional boost irradiation of 10–14 Gy/5–7 fractions was administered when the surgical margin was ≤5 mm (20, 21).

### 2.5 Definition of LRR

LRR encompassed both local recurrence and regional recurrence. Local recurrence refers to recurrence on the same side of the breast, chest wall, skin, or surgical scar. Regional recurrence refers to recurrence in the lymphatic drainage area, including the axillary, supraclavicular, internal mammary, or subclavian lymph nodes (22, 23). Tumor recurrence was evaluated through imaging examinations or, if necessary, pathological examination. Based on the above definition, the patients were divided into two groups: recurrent group and non-recurrent group.

### 2.6 Statistical analysis

The data were analyzed by R 4.3.1 software. Independent-sample t-tests, chi-square tests, or Fisher’s exact tests were used, as needed, to illustrate the clinicopathological parameters. The data set was randomly divided into training and validation sets at a 7:3 ratio. Univariate and multivariate logistic regression analysis were conducted to identify the factors influencing LRR in patients with BC. The stepwise backward regression method was employed to construct the prediction model. Odds ratios (OR) were calculated to determine effect values. To assess the accuracy and stability of the model, the confusion matrix was used to calculate the accuracy, recall, Kappa value, and F-measure in both the training and validation sets. Discrimination, calibration, and clinical practicability were evaluated to measure the model’s performance. Discrimination was assessed using the receiver operating characteristic (ROC) curve. The calibration degree was evaluated by the calibration curve and Hosmer–Lemeshow goodness of fit test. Clinical practicability was evaluated through decision curve analysis (DCA). Nomograms for predicting the risk of RR in patients with BC were drawn using the “rmda,” “rms,” and “ggplot2” R packages. Statistical significance was considered at *P*-values <0.05.

## 3 Results

### 3.1 Clinicopathological characteristics of patients

In total, 238 patients with BC were enrolled, with 118 (49.58%) patients in the recurrent group and 120 (50.42%) in the non-recurrent group. Of these, 62(26.05%) patients were classified as stage IA/IB, 69(28.99%) patients as stage IIA/IIB and 107(44.96) patients as stage IIIA/IIIB/IIIC. Additionally, 113(47.48%) patients were classified with NPI1, 95(39.92%) patients with NPI2 and 30(12.61%) patients with NPI3. In terms of subtype, 110(46.22%) patients had Luminal A cancer,49(20.59%) patients had Luminal B cancer, 45(18.91%) patients had HER2+/ER- cancer and 34(14.29%) patients had triple-negative cancer.

The mean score of NPI were (4.13 ± 1.52) and (3.11 ± 1.37) points in the recurrent and non-recurrent groups, respectively, with significant differences (t= 5.386, *P*<0.001). Regarding Eastern Cooperative Oncology Group performance (ECOG) scores, 60(25.21%) patients had an ECOG score of 0 points, 106 (44.54%) patients had a score of 1 point, and 72 (30.25%) patients had a score of 2 points. **Table 1** displays the comparative information between the two patient groups. The samples were randomly divided into a training set of 167 cases and validation set of 71 cases. There were no significant differences in any of the variables between the two sets, suggesting that the source of patients in the two groups was the same. **Table 2** shows the comparative information between the training and validation sets. The panoramic data of the patients included in the study are presented in **Supplementary Table S1**.

**Table 1:** Clinicopathological characteristics between recurrent group and non-recurrent group.

Variables	Levels	Total	Recurrent group (n=118)	Non-recurrent group (n=120)	Statistic	*P*
ECOG	ECOG 0	60(25.21)	29(24.58)	31(25.83)	1.543	0.462
	ECOG 1	106(44.54)	49(41.53)	57(47.50)		
	ECOG 2	72(30.25)	40(33.90)	32(26.67)		
Subtype	Luminal A	110(46.22)	45(38.14)	65(54.17)	7.323	0.062
	Luminal B	49(20.59)	25(21.19)	24(20.00)		
	HER2+/ER-	45(18.91)	27(22.88)	18(15.00)		
	Triple-negative	34(14.29)	21(17.80)	13(10.83)		
Chemotherapy	No	46(19.33)	18(15.25)	28(23.33)	2.491	0.115
	Yes	192(80.67)	100(84.75)	92(76.67)		
Vascular invasion	No	171(71.85)	75(63.56)	96(80.00)	7.951	0.005
	Yes	67(28.15)	43(36.44)	24(20.00)		
Perineural invasion	No	184(77.31)	83(70.34)	101(84.17)	6.485	0.011
	Yes	54(22.69)	35(29.66)	19(15.83)		
Stage	Stage I	62(26.05)	16(13.56)	46(38.33)	25.041	<0.001
	Stage II	69(28.99)	32(27.12)	37(30.83)		
	Stage III	107(44.96)	70(59.32)	37(30.83)		
NPI	NPI1	113(47.48)	36(30.51)	77(64.17)	30.304	<0.001
	NPI2	95(39.92)	58(49.15)	37(30.83)		
	NPI3	30(12.61)	24(20.34)	6(5.00)		
ER	Negative	79(33.19)	48(40.68)	31(25.83)	5.912	0.015
	Positive	159(66.81)	70(59.32)	89(74.17)		
PR	Negative	79(33.19)	48(40.68)	31(25.83)	7.299	0.026
	Low	49(20.59)	25(21.19)	24(20.00)		
	Positive	110(46.22)	45(38.14)	65(54.17)		
HER2	Negative	193(81.09)	91(77.12)	102(85.00)	2.410	0.121
	Positive	45(18.91)	27(22.88)	18(15.00)		
MKI67	Low	133(55.88)	58(49.15)	75(62.50)	4.576	0.101
	Middle	12(5.04)	6(5.08)	6(5.00)		
	High	93(39.08)	54(45.76)	39(32.50)		
Endocrine therapy	No	79(33.19)	48(40.68)	31(25.83)	5.912	0.015
	Yes	159(66.81)	70(59.32)	89(74.17)		
Tumor location	Right	112(47.06)	53(44.92)	59(49.17)	0.432	0.511
	Left	126(52.94)	65(55.08)	61(50.83)		
Tumor quadrant	Outer Upper Quadrant	100(42.02)	52(44.07)	48(40.00)	2.666	0.446
	Outer Lower Quadrant	74(31.09)	37(31.36)	37(30.83)		
	Inner Upper Quadrant	37(15.55)	14(11.86)	23(19.17)		
	Inner Lower Quadrant	27(11.34)	15(12.71)	12(10.00)		
Radiotherapy techniques	Conventional Fractionated	83(34.87)	37(31.36)	46(38.33)	1.275	0.259
	Hypofractionated	155(65.13)	81(68.64)	74(61.67)		
Grade	G1	52(21.85)	17(14.41)	35(29.17)	13.360	0.001
	G2	135(56.72)	66(55.93)	69(57.50)		
	G3	51(21.43)	35(29.66)	16(13.33)		
Tstage	T1	111(46.64)	42(35.59)	69(57.50)	15.476	0.001
	T2	69(28.99)	42(35.59)	27(22.50)		
	T3	32(13.45)	15(12.71)	17(14.17)		
	T4	26(10.92)	19(16.10)	7(5.83)		
Nstage	N0	92(38.66)	31(26.27)	61(50.83)	20.697	<0.001
	N1	68(28.57)	36(30.51)	32(26.67)		
	N2	56(23.53)	33(27.97)	23(19.17)		
	N3	22(9.24)	18(15.25)	4(3.33)		
Age		55.97 ± 8.64	58.42 ± 8.31	53.58 ± 8.30	4.495	<0.001
BMI		24.20 ± 2.29	24.62 ± 2.21	23.78 ± 2.30	2.853	0.005
MKI67.c		21.33 ± 18.14	23.82 ± 18.20	18.88 ± 17.81	2.116	0.035
Tstage.c		2.94 ± 1.92	3.26 ± 1.91	2.61 ± 1.88	2.634	0.009
NPI.c		3.62 ± 1.53	4.13 ± 1.52	3.11 ± 1.37	5.386	<0.001
Nstage.c		3.35 ± 4.22	4.48 ± 4.74	2.24 ± 3.30	4.228	<0.001

ECOG, Eastern Cooperative Oncology Group performance; ER, refers to estrogen receptor; PR, refers to progesterone receptor; MKI67, Ki-67 index; MKI67.c, continuous data of MKI67 (Ki-67%); Tstage.c, tumor size of primary site; NPI.c, continuous data of; Nstage.c, number of positive lymph nodes; BMI, body mass index.

**Table 2:** Clinicopathological characteristics between training set and validation set.

Variables	Levels	Total	Training set (n=167)	Validation set (n=71)	Statistic	*P*
ECOG	ECOG 0	60(25.21)	40(23.95)	20(28.17)	1.096	0.578
	ECOG 1	106(44.54)	78(46.71)	28(39.44)		
	ECOG 2	72(30.25)	49(29.34)	23(32.39)		
Subtype	Luminal A	110(46.22)	81(48.50)	29(40.85)	1.526	0.676
	Luminal B	49(20.59)	33(19.76)	16(22.54)		
	HER2+/ER-	45(18.91)	29(17.37)	16(22.54)		
	Triple-negative	34(14.29)	24(14.37)	10(14.08)		
Chemotherapy	No	46(19.33)	35(20.96)	11(15.49)	0.954	0.329
	Yes	192(80.67)	132(79.04)	60(84.51)		
Vascular invasion	No	171(71.85)	123(73.65)	48(67.61)	0.901	0.343
	Yes	67(28.15)	44(26.35)	23(32.39)		
Perineural invasion	No	184(77.31)	133(79.64)	51(71.83)	1.732	0.188
	Yes	54(22.69)	34(20.36)	20(28.17)		
Stage	Stage I	62(26.05)	46(27.54)	16(22.54)	1.397	0.497
	Stage II	69(28.99)	50(29.94)	19(26.76)		
	Stage III	107(44.96)	71(42.51)	36(50.70)		
NPI	NPI1	113(47.48)	80(47.90)	33(46.48)	0.203	0.904
	NPI2	95(39.92)	67(40.12)	28(39.44)		
	NPI3	30(12.61)	20(11.98)	10(14.08)		
ER	Negative	79(33.19)	53(31.74)	26(36.62)	0.536	0.464
	Positive	159(66.81)	114(68.26)	45(63.38)		
PR	Negative	79(33.19)	53(31.74)	26(36.62)	1.176	0.555
	Low	49(20.59)	33(19.76)	16(22.54)		
	Positive	110(46.22)	81(48.50)	29(40.85)		
HER2	Negative	193(81.09)	138(82.63)	55(77.46)	0.868	0.351
	Positive	45(18.91)	29(17.37)	16(22.54)		
Variables	Levels	Total	Training set (n=167)	Validation set (n=71)	Statistic	*P*
MKI67	Low	133(55.88)	92(55.09)	41(57.75)	0.289	0.865
	Middle	12(5.04)	8(4.79)	4(5.63)		
	High	93(39.08)	67(40.12)	26(36.62)		
Endocrine therapy	No	79(33.19)	53(31.74)	26(36.62)	0.536	0.464
	Yes	159(66.81)	114(68.26)	45(63.38)		
Tumor location	Right	112(47.06)	78(46.71)	34(47.89)	0.028	0.867
	Left	126(52.94)	89(53.29)	37(52.11)		
Tumor quadrant	Outer Upper Quadrant	100(42.02)	73(43.71)	27(38.03)	0.936	0.817
	Outer Lower Quadrant	74(31.09)	51(30.54)	23(32.39)		
	Inner Upper Quadrant	37(15.55)	24(14.37)	13(18.31)		
	Inner Lower Quadrant	27(11.34)	19(11.38)	8(11.27)		
Radiotherapy techniques	Conventional Fractionated	83(34.87)	58(34.73)	25(35.21)	0.005	0.943
	Hypofractionated	155(65.13)	109(65.27)	46(64.79)		
Grade	G1	52(21.85)	35(20.96)	17(23.94)	0.265	0.876
	G2	135(56.72)	96(57.49)	39(54.93)		
	G3	51(21.43)	36(21.56)	15(21.13)		
Tstage	T1	111(46.64)	85(50.90)	26(36.62)	4.535	0.209
	T2	69(28.99)	43(25.75)	26(36.62)		
	T3	32(13.45)	21(12.57)	11(15.49)		
	T4	26(10.92)	18(10.78)	8(11.27)		
Nstage	N0	92(38.66)	66(39.52)	26(36.62)	1.124	0.771
	N1	68(28.57)	47(28.14)	21(29.58)		
	N2	56(23.53)	37(22.16)	19(26.76)		
	N3	22(9.24)	17(10.18)	5(7.04)		
Age		55.97 ± 8.64	55.89 ± 7.95	56.01 ± 8.93	0.107	0.915
BMI		24.20 ± 2.29	24.10 ± 2.18	24.24 ± 2.34	0.446	0.656
MKI67.c		21.33 ± 18.14	21.00 ± 18.17	21.47 ± 18.17	0.184	0.855
Tstage.c		2.94 ± 1.92	3.20 ± 1.86	2.82 ± 1.94	1.412	0.160
NPI.c		3.62 ± 1.53	3.65 ± 1.48	3.60 ± 1.56	0.252	0.801
Nstage.c		3.35 ± 4.22	3.31 ± 3.93	3.37 ± 4.35	0.107	0.915

**Figure 2:**
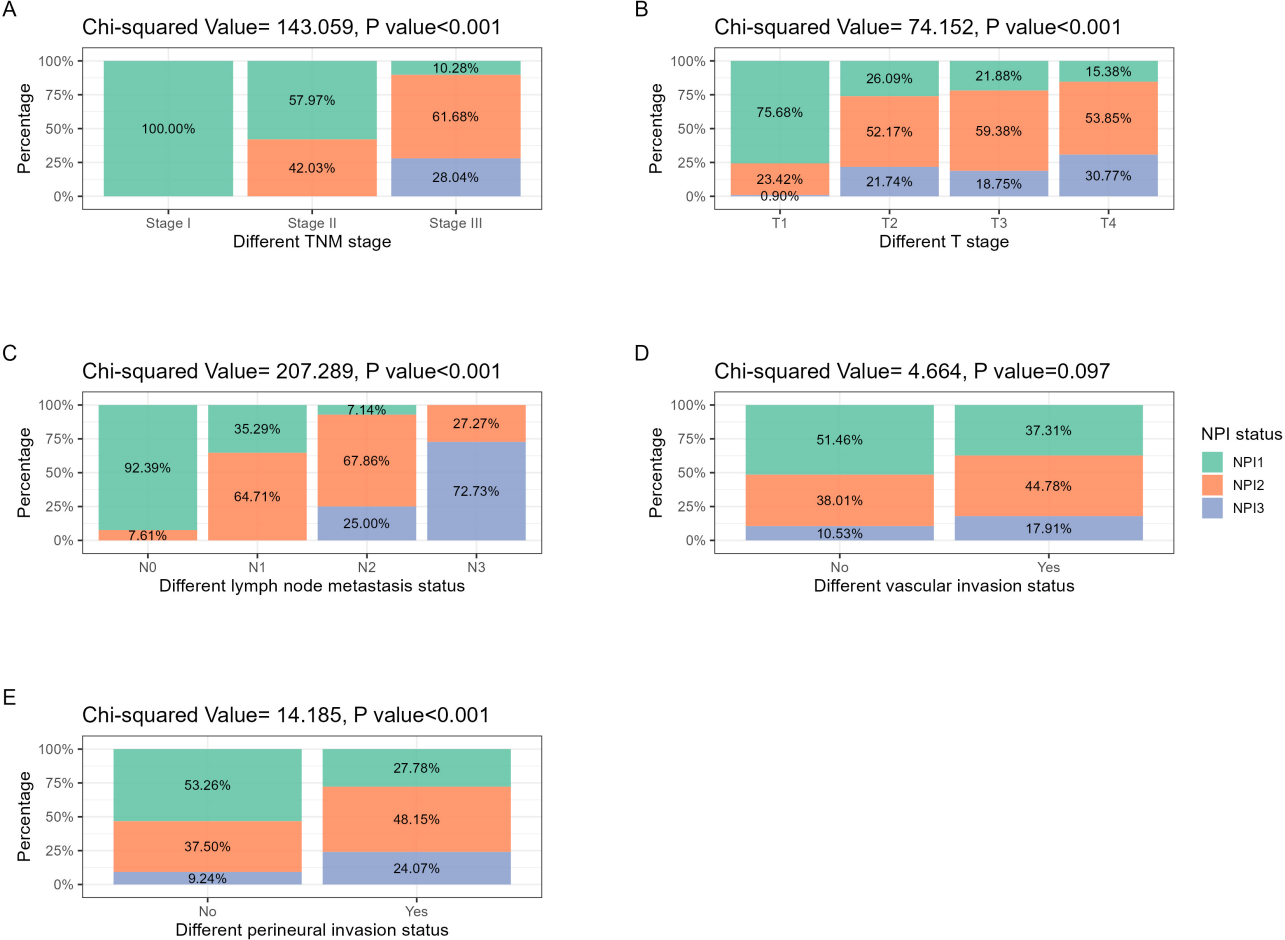
NPI distribution in different pathological features related to tumor stage and invasion. **(A)** TNM stage: with the increase of tumor TNM stage, NPI grade increased significantly (*P*<0.001). **(B)** T stage: with the increasing extent of tumor invasion, the NPI grade increased significantly (*P*<0.001). **(C)** N stage: with the increase of the number of tumor lymph node metastasis, the NPI grade increased significantly (*P*<0.001). **(D)** Vascular invasion: in the population with positive vascular invasion, the proportion of high-grade NPI tended to increase, but the difference was not statistically significant (*P*<0.097). **(E)** Perineural invasion: in the population with positive perineural invasion, the proportion of high-grade NPI increased significantly (*P*<0.001).

In addition, considering that TNM stage and NPI were both related to T stage and N stage, correlation analysis for some significant pathological parameters was conducted via Chi square test, and the results were shown in **Figure 2**. In addition to vascular invasion, NPI was significantly correlated with tumor stage, T stage, N stage, and perineural invasion(*P*<0.001).

### 3.2 Univariate and multivariate logistic regression analysis of NPI scores

**Figure 3:**
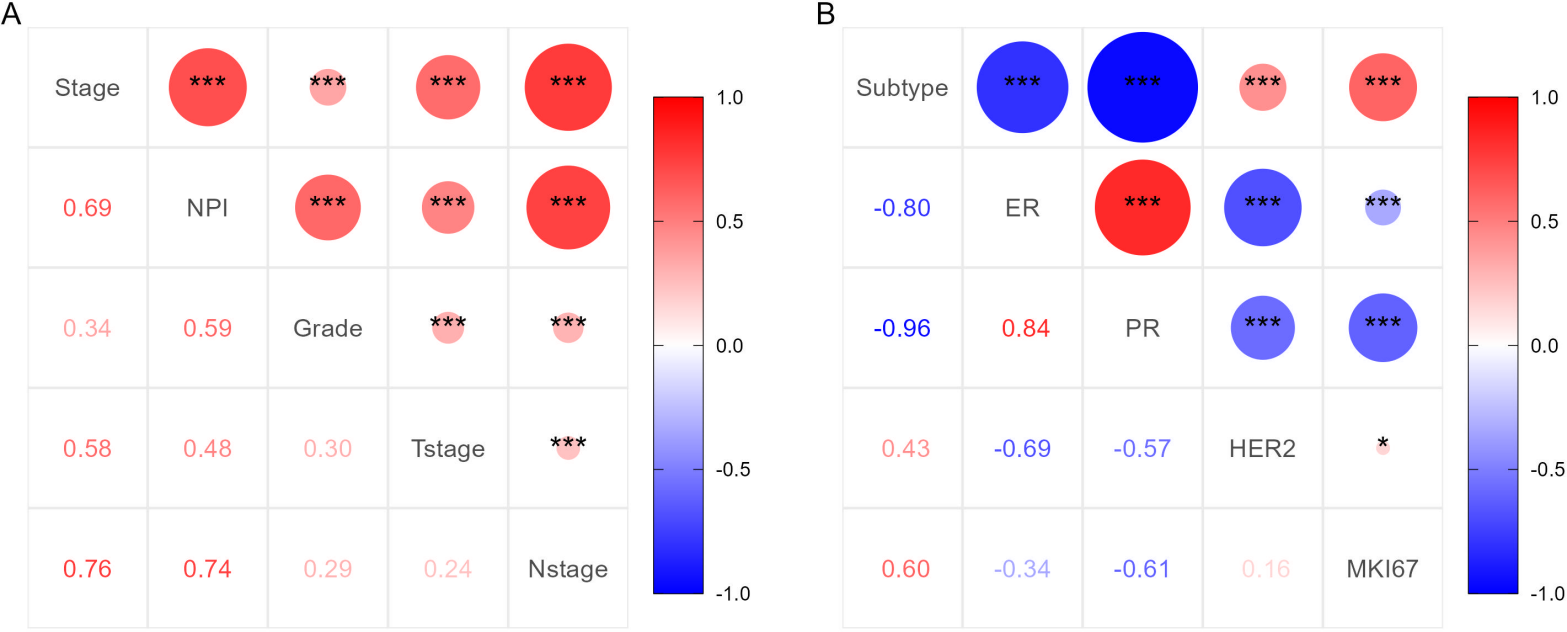
Correlation analysis results of important clinicopathological features. **(A)** Correlation analysis results for TNM stage, NPI, T stage, N stage and tumor grade. **(B)** Correlation analysis results for subtype of BC, ER, PR, HER2 and Ki-67.

Before conducting multivariate logistic regression modeling, we performed correlation analysis on certain variables to account for collinearity in the model. Initially, we examined the correlation between tumor TNM stage, NPI, T stage, N stage, and tumor grade. The results indicated a strong correlation between tumor TNM stage and NPI with T stage, N stage, and tumor grade (*P <*0.001). This suggested that T stage, N stage, and tumor grade should be excluded from the multivariable model, and tumor TNM stage and NPI should be modeled separately. The correlation analysis results are shown in **Figure 3A**. Additionally, we analyzed the correlations among ER, PR, HER2, and Ki-67 to determine the pathological subtype of BC. The results displayed a significant correlation between the subtypes of BC cases and ER, PR, HER2, and Ki-67 (P <0.001). Thus, ER, PR, HER2, and Ki-67 were excluded from the multivariate model. These results are presented in **Figure 3B**. Subsequently, univariate logistic regression analysis was conducted to explore potential risk factors for LRR using the training samples. Age, BMI, TNM stage, NPI, vascular invasion, and perineural invasion were identified as potential risk factors for LRR (P <0.05) (**Table 3**).

**Table 3:** Univariate analysis results of radioresistance from logistic regression model.

Variables	Levels	Beta	Standard error	OR	Z value	*P*
Age		0.06	0.02	1.06(1.02,1.10)	3.136	0.002
BMI		0.27	0.07	1.30(1.13,1.50)	3.675	<0.001
Stage	Stage I					
	Stage II	0.72	0.44	2.05(0.86,4.87)	1.628	0.103
	Stage III	1.91	0.42	6.75(2.93,15.51)	4.495	<0.001
NPI	NPI1					
	NPI2	1.25	0.35	3.49(1.77,6.90)	3.596	<0.001
	NPI3	1.83	0.57	6.23(2.04,19.00)	3.216	0.001
Subtype	Luminal A					
	Luminal B	0.38	0.41	1.47(0.65,3.31)	0.927	0.354
	HER2+/ER-	0.82	0.44	2.26(0.95,5.40)	1.838	0.066
	Triple-negative	0.66	0.47	1.94(0.77,4.87)	1.401	0.161
Vascular invasion	No					
	Yes	1.18	0.38	3.26(1.55,6.82)	3.127	0.002
Perineural invasion	No					
	Yes	0.93	0.41	2.54(1.15,5.64)	2.300	0.021
Chemotherapy	No					
	Yes	0.65	0.39	1.91(0.89,4.11)	1.656	0.098
Endocrine therapy	No					
	Yes	-0.63	0.34	0.53(0.27,1.03)	1.871	0.061
Tumor location	Right					
	Left	0.07	0.31	1.08(0.59,1.98)	0.238	0.812
Tumor quadrant	Outer Upper Quadrant					
	Outer Lower Quadrant	-0.31	0.37	0.73(0.36,1.50)	0.847	0.397
	Inner Upper Quadrant	-0.89	0.49	0.41(0.16,1.08)	1.797	0.072
	Inner Lower Quadrant	0.13	0.52	1.13(0.41,3.15)	0.242	0.809
Radiotherapy techniques	Conventional Fractionated					
	Hypofractionated	-0.12	0.33	0.88(0.47,1.67)	0.381	0.703
ECOG	ECOG 0					
	ECOG 1	-0.46	0.39	0.63(0.29,1.36)	1.172	0.241
	ECOG 2	0.00	0.43	1.00(0.43,2.33)	0.010	0.992

**Figure 4:**
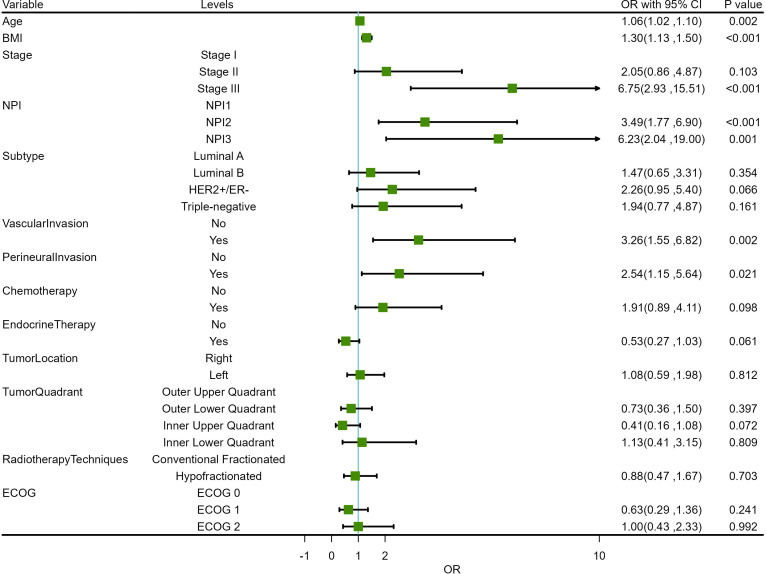
Forest plots for univariate regression analysis of risk factors.

**Figure 5:**
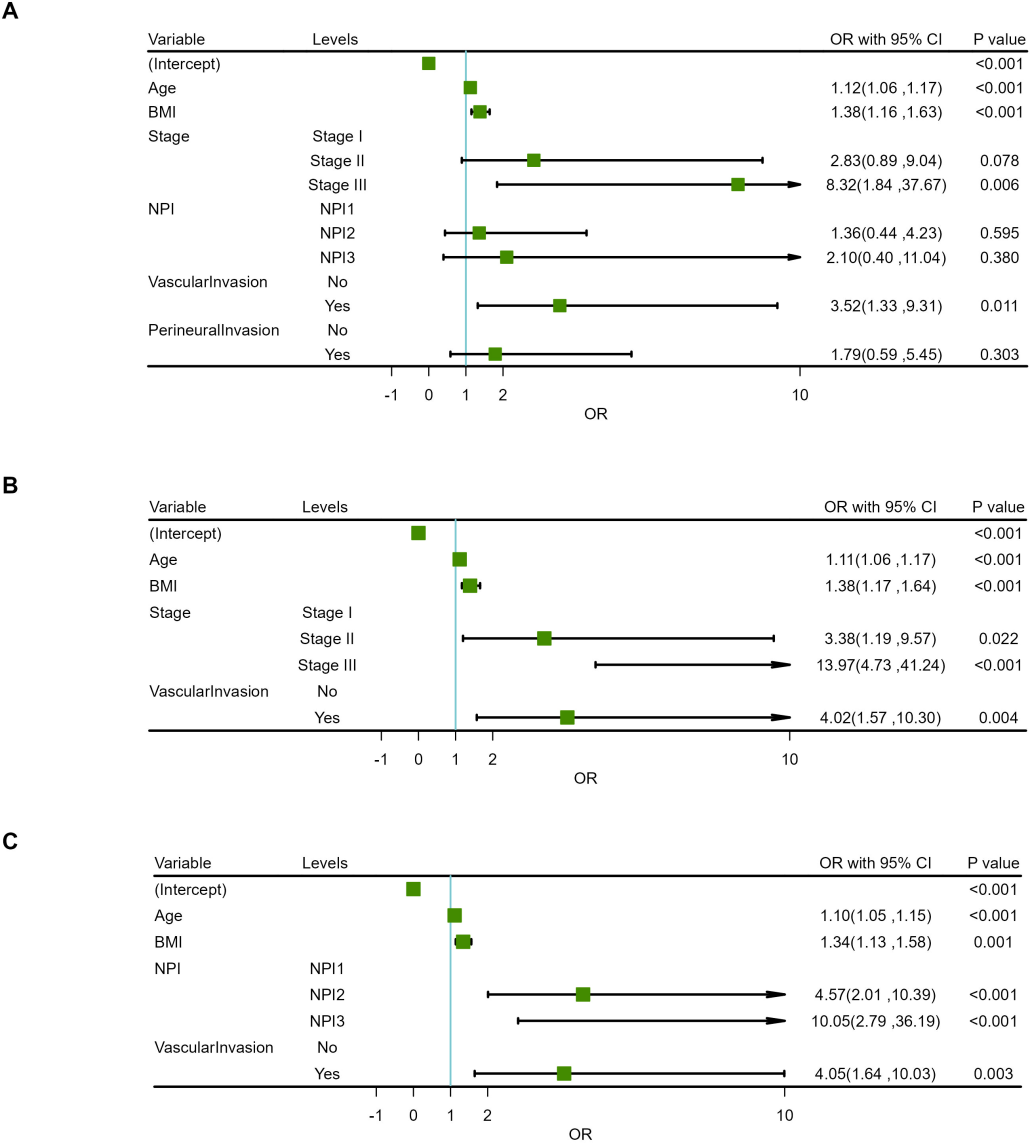
Forest plots for multivariate regression analysis of risk factors. **(A)** 6-variable model. **(B)** TNM-stage-based model. **(C)** NPI-based model.

Based on the positive variables obtained from the univariate analysis, a multivariate logistic regression analysis was performed, resulting in a six-variable model (**Table 4**). In this model, the effect of the NPI was counteracted, and the effect of perineural invasion disappeared, suggesting that perineural invasion is not an independent risk factor for LRR. After conducting a backward stepwise regression analysis, four variables were found to have independent risk effects on LRR: age, BMI, vascular invasion, and TNM stage. Consequently, a prediction model based solely on TNM stage was developed (TNM-stage-based model) (**Table 5**). To assess the predictive effect of the NPI on LRR, TNM stage was removed from the six-variable model, resulting in an NPI-based model (**Table 6**). All predictors in the NPI-based model were found to have significant effects on LRR (P <0.05). Forest plots depicting the results of the univariate and multivariate regression analyses of risk factors are presented in **Figures 4**, **5A–C**.

**Table 4:** Multivariate analysis results of locoregional recurrence from logistic regression model (6-variate model).

Variables	Levels	Beta	Standard error	OR	Z value	*P*
Intercept		-15.75	2.84		5.540	<0.001
Age		0.11	0.03	1.12(1.06,1.17)	4.250	<0.001
BMI		0.32	0.09	1.38(1.16,1.63)	3.690	<0.001
Stage	Stage I					
	Stage II	1.04	0.59	2.83(0.89,9.04)	1.761	0.078
	Stage III	2.12	0.77	8.32(1.84,37.67)	2.749	0.006
NPI	NPI1					
	NPI2	0.31	0.58	1.36(0.44,4.23)	0.531	0.595
	NPI3	0.74	0.85	2.10(0.40,11.04)	0.877	0.380
Vascular invasion	No					
	Yes	1.26	0.50	3.52(1.33,9.31)	2.539	0.011
Perineural invasion	No					
	Yes	0.58	0.57	1.79(0.59,5.45)	1.029	0.303

all significant variates from univariate analysis were included in this 6-variate model.

**Table 5:** Multivariate analysis results of locoregional recurrence from logistic regression model (TNM-stage-based model).

Variables	Levels	Beta	Standard error	OR	Z value	*P*
Intercept		-15.57	2.82		5.529	<0.001
Age		0.10	0.03	1.11(1.06,1.17)	4.152	<0.001
BMI		0.33	0.09	1.38(1.17,1.64)	3.785	<0.001
Stage	Stage I					
	Stage II	1.22	0.53	3.38(1.19,9.57)	2.291	0.022
	Stage III	2.64	0.55	13.97(4.73,41.24)	4.775	<0.001
Vascular invasion	No					
	Yes	1.39	0.48	4.02(1.57,10.30)	2.900	0.004

**Table 6:** Multivariate analysis results of locoregional recurrence from logistic regression model (NPI-based model).

Variables	Levels	Beta	Standard error	OR	Z value	*P*
Intercept		-13.67	2.60		5.248	<0.001
Age		0.10	0.02	1.10(1.05,1.15)	4.022	<0.001
BMI		0.29	0.08	1.34(1.13,1.58)	3.464	0.001
NPI	NPI1					
	NPI2	1.52	0.42	4.57(2.01,10.39)	3.627	<0.001
	NPI3	2.31	0.65	10.05(2.79,36.19)	3.531	<0.001
Vascular invasion	No					
	Yes	1.40	0.46	4.05(1.64,10.03)	3.023	0.003

### 3.3 Establishment of a prediction model based on logistic regression

**Figure 6:**
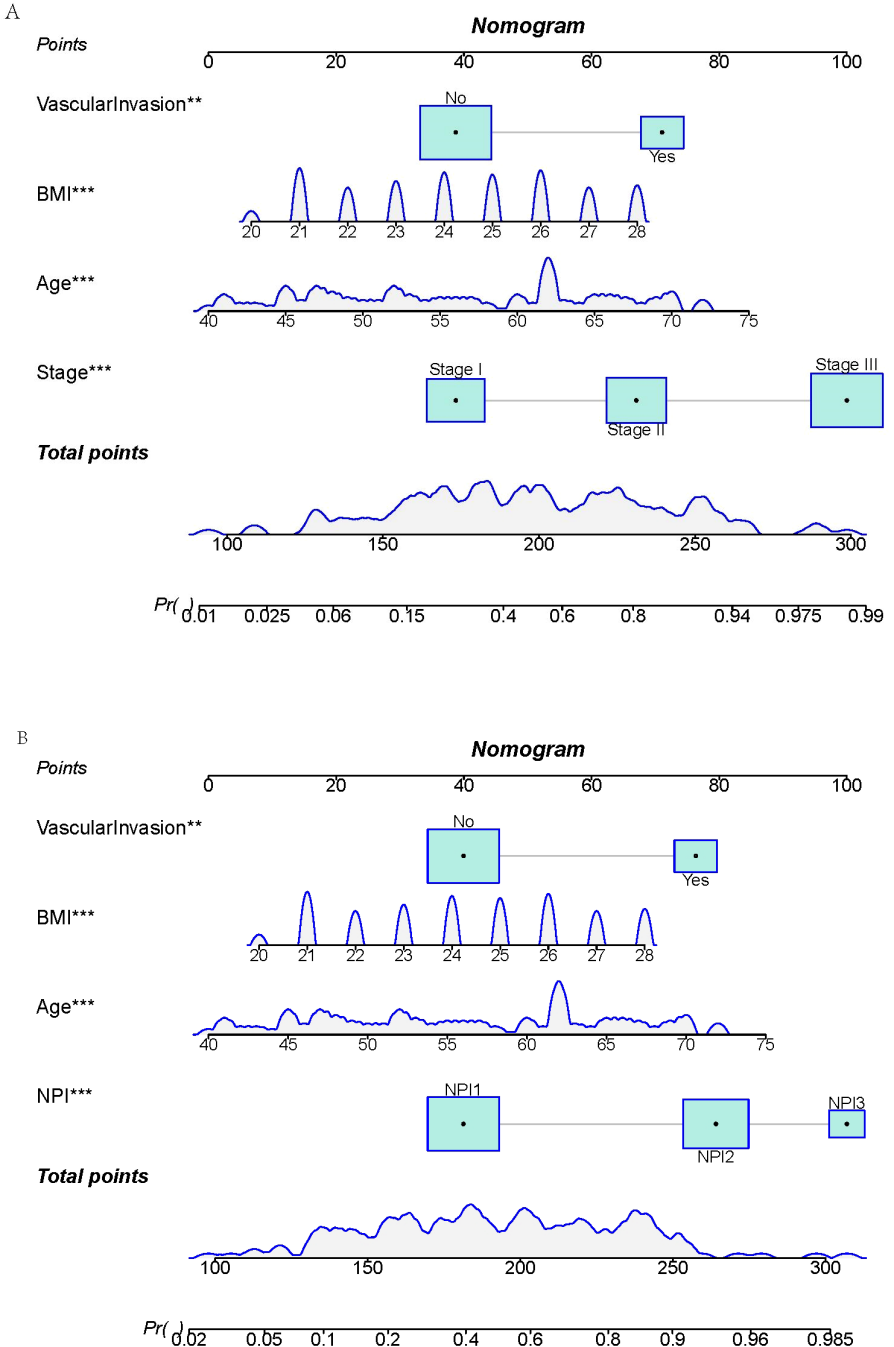
Nomograms for predicting locoregional recurrence in breast cancer. **(A)** TNM-stage-based model. **(B)** NPI-based model.

Two prediction models were developed based on the parameters in **Tables 5**, **6**. The TNM-stage-based model was calculated according to the following formula: [-15.57+(0.10×Age) + (0.33×BMI) + (1.22×Stage (Stage II)) +(2.64×Stage (Stage III)) + (1.39×VascularInvasion (Yes))]. Meanwhile, the NPI-based model was calculated according to the following formula: [-13.67 + (0.10×Age) + (0.29×BMI) + (1.52×NPI (NPI2)) + (2.31×NPI (NPI3)) + (1.40×VascularInvasion (Yes))]. Using the coefficients from the multivariate logistic regression model, two predictive nomograms of LRR (**Figures 6A, B**) were created using the “rms” package in R. These nomograms comprise seven axes in total, with two to six axes representing five variables in the predictive model. The estimated score of each risk factor can be calculated by drawing a line perpendicular to the corresponding axis. The total score is then obtained by summing these individual scores, which is used to predict the probability of predicting LRR following ART.

### 3.4 Model evaluation

#### 3.4.1 Accuracy evaluation of the models

**Figure 7:**
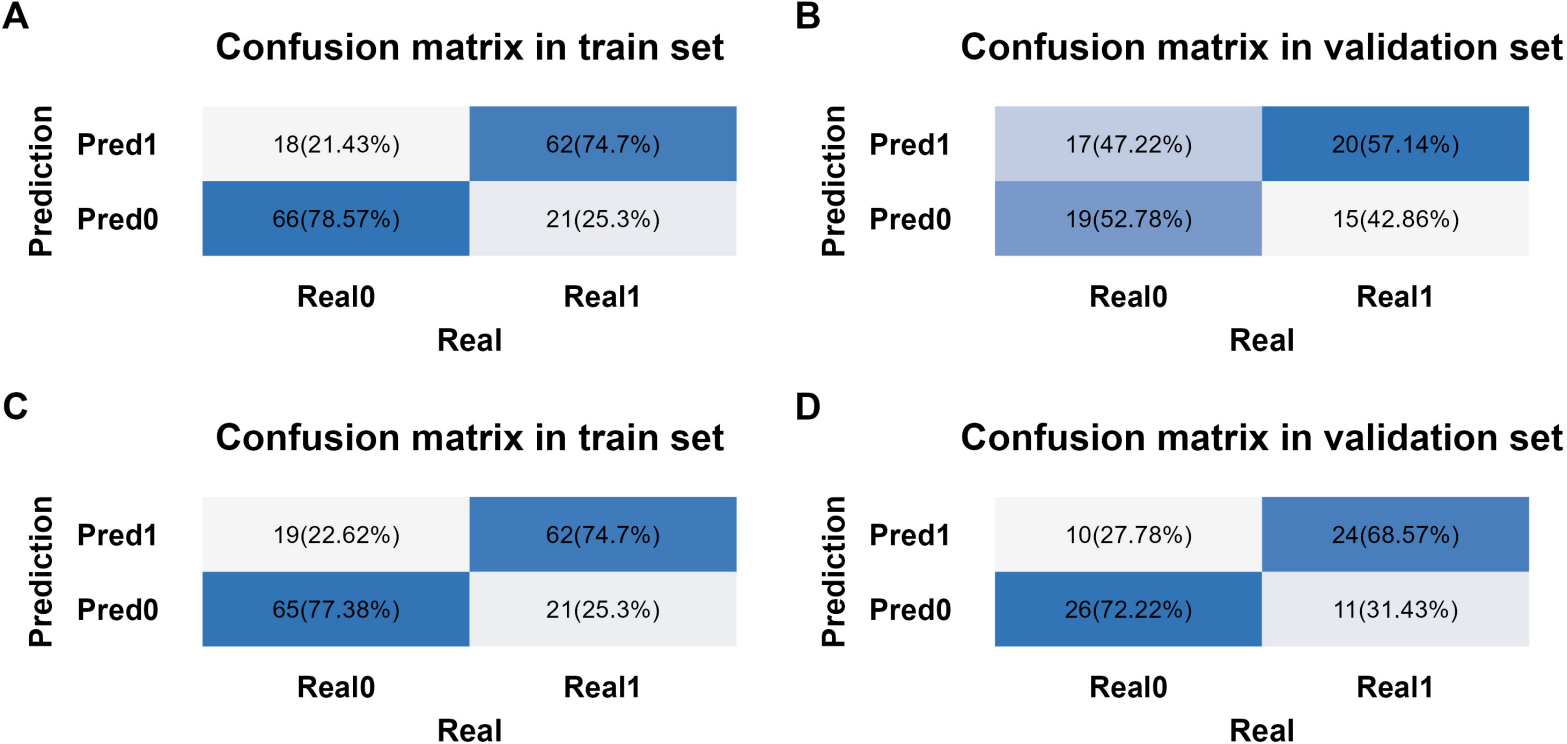
Confusion matrix analysis in different models and datasets. **(A)** Confusion matrix from training set in TNM-stage-based model. **(B)** Confusion matrix from validation set in TNM-stage-based model. **(C)** Confusion matrix from training set in NPI-based model. **(D)** Confusion matrix from validation set in NPI-based model.

The Akaike information criterion (AIC) values of three multivariate prediction models are shown in **Supplementary Table S2**. The confusion matrices for the TNM-stage-based model and NPI-based model in training set and validation set are shown in **Figures 7A–D**. With these confusion matrices, accuracy evaluation indicators of the models were calculated and presented in **Table 7**.

**Table 7:** Accuracy evaluation of the models.

Indicators	TNM-stage-based model		NPI-based model	
	Training set	Validation set	Training set	Validation set
Cox-Snell R-Squared	0.337	0.620	0.304	0.574
Nagelkerke R-Squared	0.450	0.645	0.405	0.597
Area under curve	0.843(0.785,0.901)	0.649(0.520,0.778)	0.830(0.766,0.893)	0.728(0.610,0.846)
Recall	0.747(0.653,0.841)	0.571(0.407,0.735)	0.747(0.653,0.841)	0.686(0.532,0.840)
F-Measure	0.761	0.556	0.756	0.696
Accuracy	0.766(0.764,0.769)	0.549(0.542,0.556)	0.760(0.758,0.763)	0.704(0.698,0.710)
Sensitivity	0.747(0.653,0.841)	0.571(0.407,0.735)	0.747(0.653,0.841)	0.686(0.532,0.840)
Specificity	0.786(0.698,0.873)	0.528(0.365,0.691)	0.774(0.684,0.863)	0.722(0.576,0.869)
Positive likelihood ratio	3.486(2.272,5.349)	1.210(0.772,1.896)	3.302(2.181,5.001)	2.469(1.393,4.376)
Negative likelihood ratio	0.322(0.219,0.474)	0.812(0.497,1.328)	0.327(0.222,0.482)	0.435(0.256,0.739)
Positive predictive value	0.775(0.683,0.867)	0.541(0.380,0.701)	0.765(0.673,0.858)	0.706(0.553,0.859)
Negative predictive value	0.759(0.669,0.849)	0.559(0.392,0.726)	0.756(0.665,0.847)	0.703(0.555,0.850)
Percentage of positive accordance	0.747(0.653,0.841)	0.571(0.407,0.735)	0.747(0.653,0.841)	0.686(0.532,0.840)
Percentage of negative accordance	0.786(0.698,0.873)	0.528(0.365,0.691)	0.774(0.684,0.863)	0.722(0.576,0.869)
Percentage of total accordance	0.766(0.702,0.831)	0.549(0.434,0.665)	0.760(0.696,0.825)	0.704(0.598,0.810)
Kappa	0.533(0.405,0.661)	0.099(-0.132,0.330)	0.521(0.391,0.650)	0.408(0.196,0.620)
Youden index	0.533	0.099	0.521	0.408

**Figure 8:**
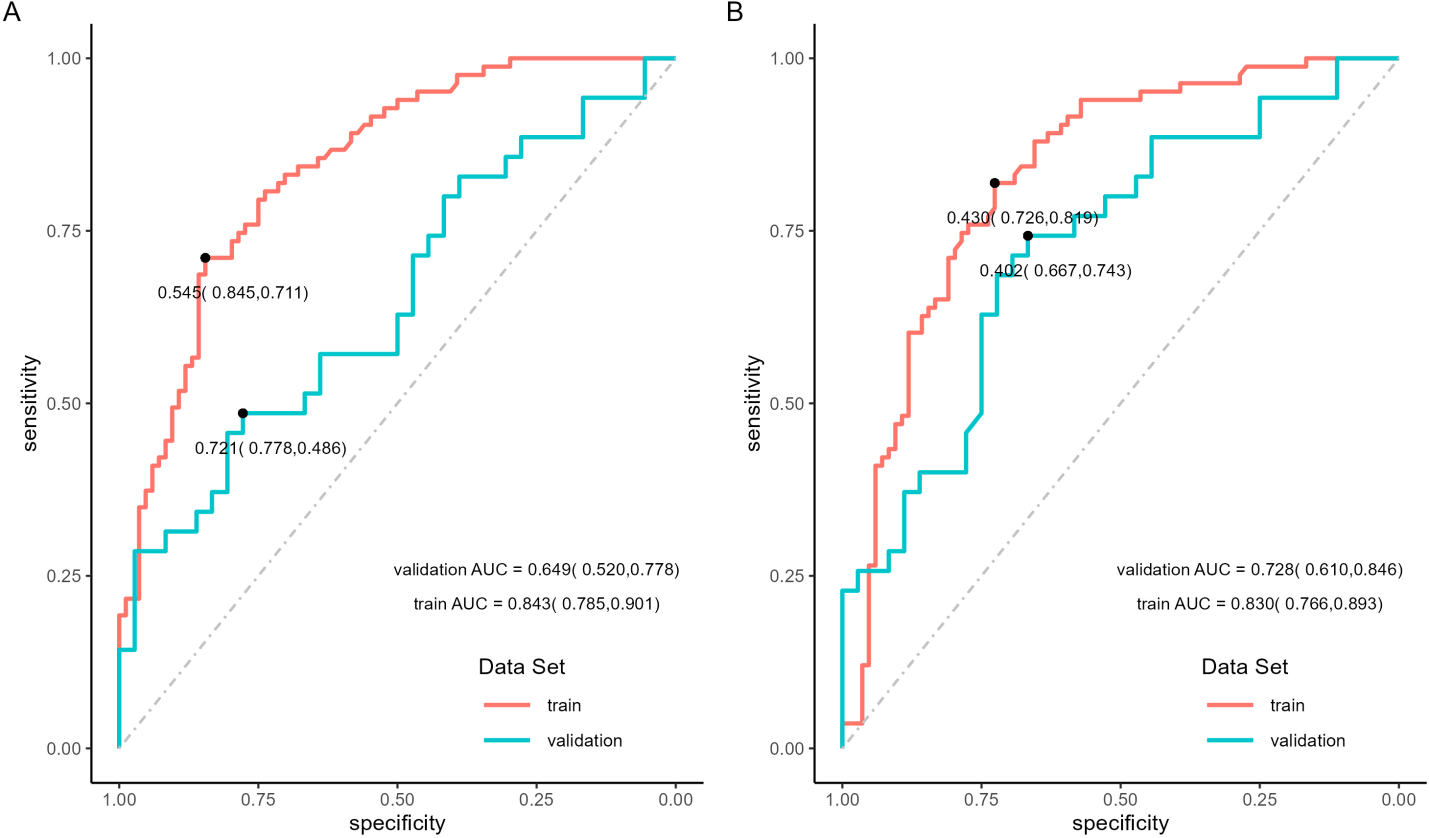
ROC curves of locoregional recurrence predictive model. **(A)** ROC curves of TNM-stage-based model in training set and validation set. **(B)** ROC curves of NPI-based model in training set and validation set.

The binary logistic regression analysis revealed that the model fit for the TNM-stage- and NPI-based models was as follows: in the training set, Cox and Snell R² values were 0.337 vs 0.304 and Nagelkerke R² values were 0.450 vs 0.405; in the validation set, Cox and Snell R² values were 0.620 vs 0.574 and Nagelkerke R² values were 0.645 vs 0.597. This indicates that both models had a good fit. In the training set, the TNM-stage- and NPI-based models demonstrated similar prediction efficiency. The area under the ROC curve (AUC) was 0.843 (95% confidence interval = (0.785, 0.901) vs 0.830 (0.766, 0.893), the accuracy was 0.766 (0.764, 0.769) vs 0.760 (0.758, 0.763), the sensitivity was 0.747 (0.653, 0.841) vs 0.747 (0.653, 0.841), and the specificity was 0.786 (0.698, 0.873) vs 0.774 (0.684, 0.863) (**Table 7**, **Figures 8A, B**). However, in the validation set, the NPI-based model had higher and more robust prediction accuracy compared to the TNM-stage-based model. The AUC was 0.649 (0.520, 0.778) vs 0.728 (0.610, 0.846), the accuracy was 0.549 (0.542, 0.556) vs 0.704 (0.698, 0.710), the sensitivity was 0.571 (0.407, 0.735) vs 0.686 (0.532, 0.840), and the specificity was 0.528 (0.365, 0.691) vs 0.722 (0.576, 0.869) (**Table 7**, **Figures 8A, B**). The F-measure and Kappa values further indicated that the NPI-based model performed significantly better.

**Figure 9:**
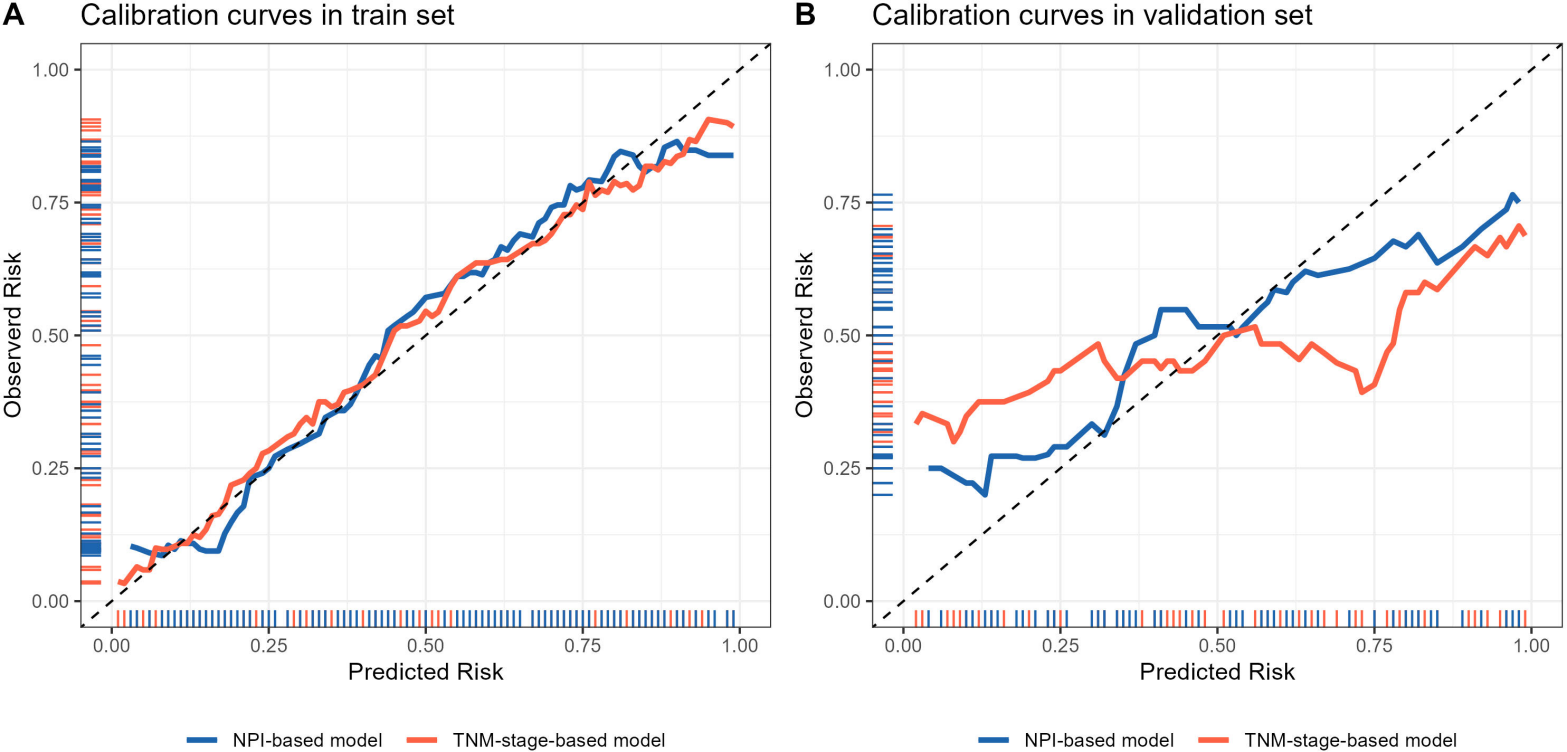
Calibration curve of locoregional recurrence prediction model. **(A)** Comparison of calibration curves of two prediction models in the training set. **(B)** Comparison of calibration curves of two prediction models in the validation set. The x-axis represents the probability of locoregional recurrence occurrence predicted by the model, and the y-axis represents the probability of actual occurrence of locoregional recurrence. The black thin dotted line represents the ideal curve, the blue and orange solid line represents the actual occurrence curve of locoregional recurrence.

The calibration curve was used to evaluate the goodness of fit of the model, and the results showed that both models had good consistency between the actual and predicted LRR risks in the training set (**Figure 9A**). However, the TNM-stage-based model had a more serious deviation than the NPI-based model in the validation set (**Figure 9B**). Hosmer-Lemeshow test was used to evaluate the calibration ability, and the bootstrap (b=500) resampling method was used for internal verification. The Hosmer-Lemeshow test results of TNM-stage-based model was χ^2^ = 11.873, df = 13, *P* = 0.538 in the training cohort, and χ^2^ = 27.287, df = 5, *P <*0.001 in the validation cohort. The Hosmer-Lemeshow test results of the NPI-based model was χ^2^ = 10.697, df = 13, *P* = 0.6362 in the training cohort, and χ^2^ = 10.105, df = 5, *P* = 0.07233 in the validation cohort. The Hosmer-Lemeshow test results indicated that there was no significant difference between the predicted probabilities and actual observed probabilities for the NPI-based model.

#### 3.4.2 Clinical application of prediction model

**Figure 10:**
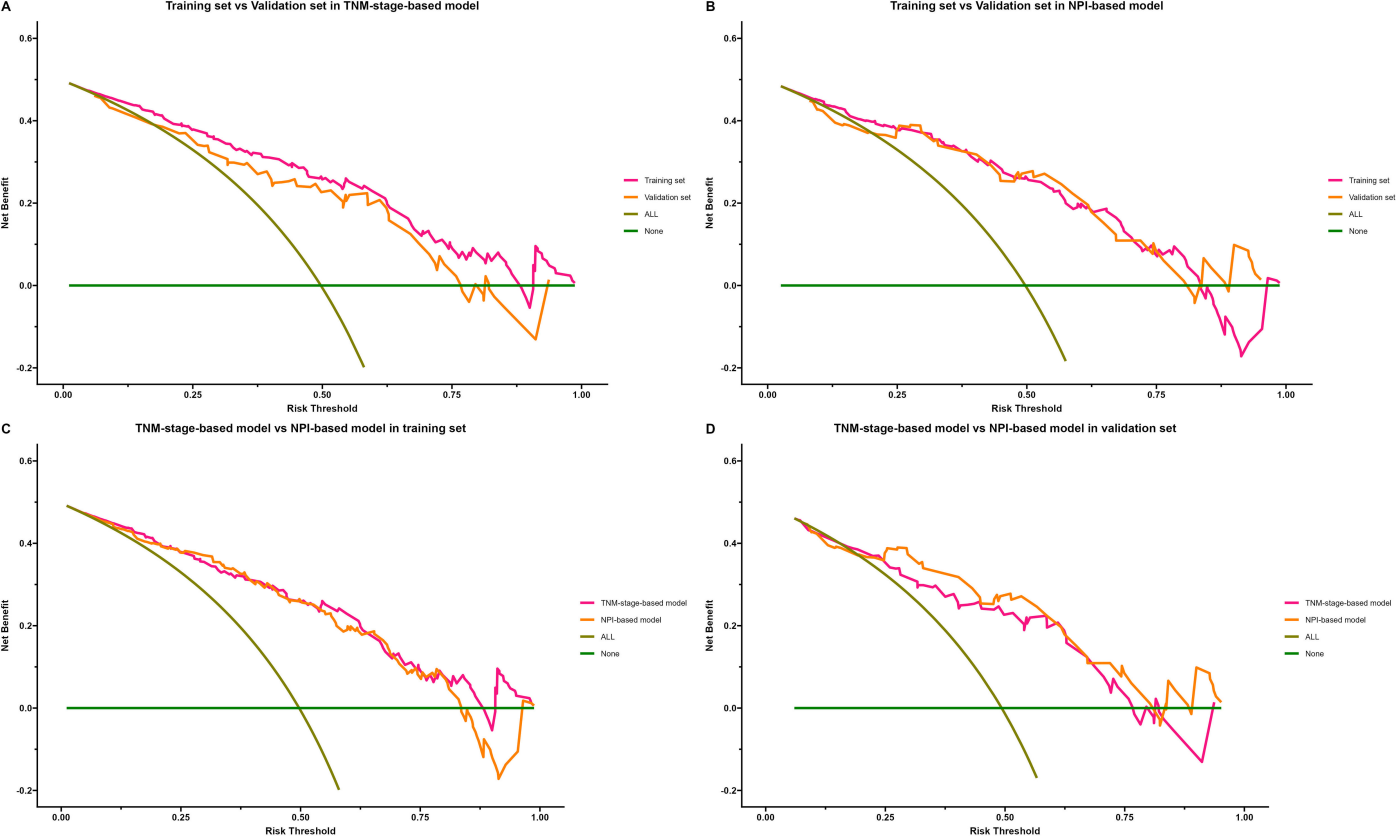
Decision curve analysis (DCA) of locoregional recurrence prediction model. The x-axis represents the threshold probability of patients diagnosed with locoregional recurrence, and the y-axis represents the net benefit rate. The green solid line represents the extreme case of assuming that neither the training sample nor the validation sample has locoregional recurrence patients, that is, the net benefit rate is zero. The brown diagonal line represents the other extreme case that all samples in training set or validation set are diagnosed as locoregional recurrence patients, and the net benefit rate is maximized. The orange and purple lines indicate the actual benefit of different cohort of patients. **(A)** Comparison of DCA of TNM-stage-based model in training set and validation set. **(B)** Comparison of DCA of NPI-based model in training set and validation set. **(C)** Comparison of DCA of TNM-stage-based model with NPI-based model in training set. **(D)** Comparison of DCA of TNM-stage-based model with NPI-based model in validation set.

**Figure 11:**
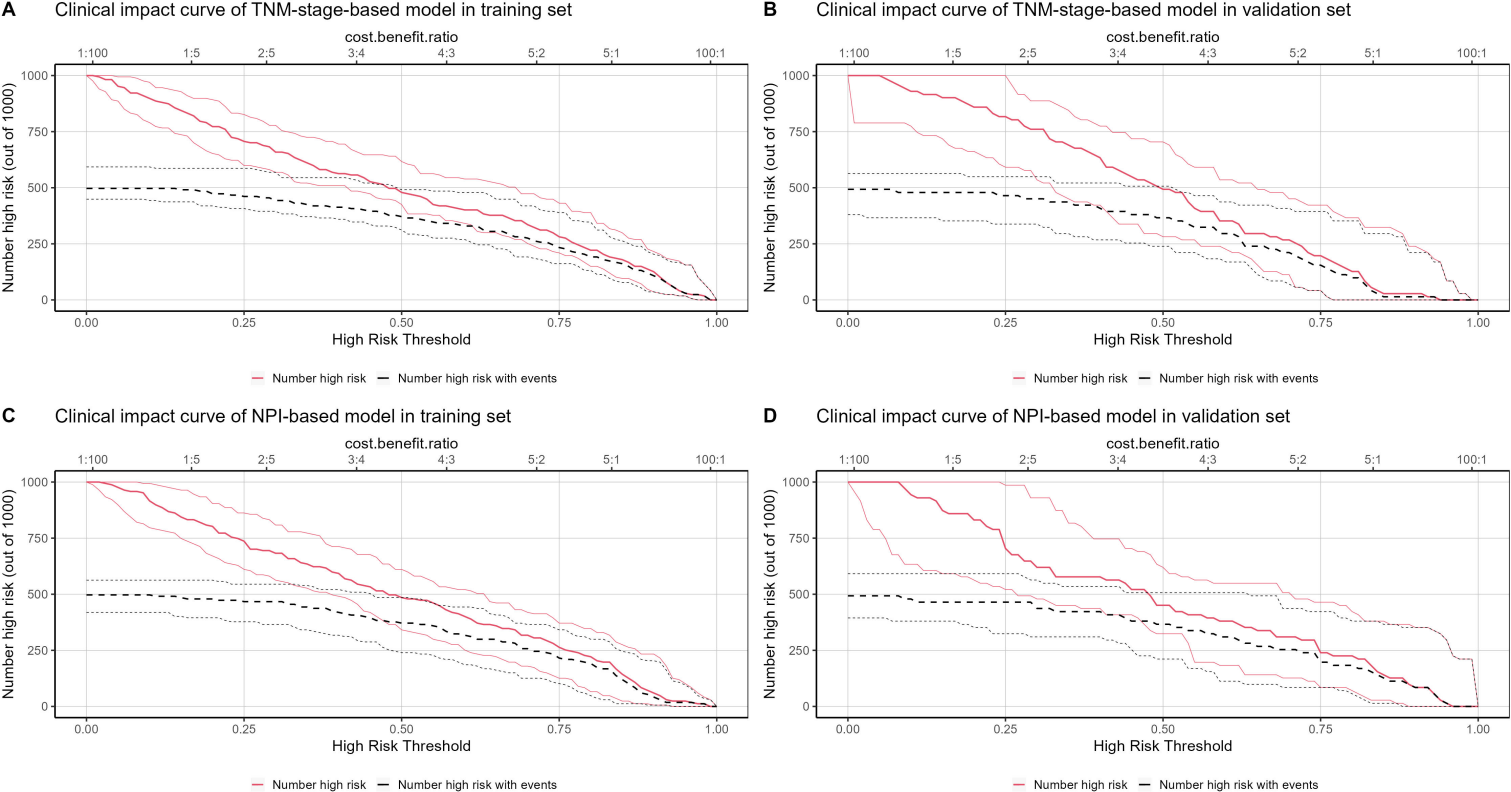
Clinical impact curve (CIC) of locoregional recurrence prediction model. The x-axis represents the threshold probability of patients diagnosed with locoregional recurrence, and the y-axis represents the number of high-risk people per 1000 people. The red line represents the number of people predicted by the model to have a locoregional recurrence event on different threshold probabilities, and the black curve represents the number of people with actual locoregional recurrence event predicted by the model to have a locoregional recurrence event on different threshold probabilities. **(A)** CIC of TNM-stage-based model in training set. **(B)** CIC of TNM-stage-based model in validation set. **(C)** CIC of NPI-based model in training set. **(D)** CIC of NPI-based model in validation set.

The clinical application of the prediction model was evaluated using DCA (**Figures 10A–D**). As shown in **Figures 10A, B**, the results of DCA indicated that the TNM-stage-based model in the training set produced a larger net benefit and wider threshold range compared to the validation set. However, the net benefit and threshold range of the NPI-based model were consistent between the training and validation sets. Turning to the results in **Figures 10C, D**, it can be observed that the performance of both the TNM-stage- and NPI-based models were highly consistent in the training set. However, in the validation set, the net benefit of the NPI-based model was slightly higher than that of the TNM-stage-based model. Overall, both models demonstrated a high net benefit for predicting the risk of LRR after ART, indicating their clinical usefulness when a median threshold of 0.5 is used as a reference. Furthermore, by analyzing the clinical impact curve (**Figures 11A–D**) in conjunction with the clinical decision curve, it can be concluded that both models exhibit better clinical efficacy and net benefit when a risk threshold of 0.5 is used as a reference. This finding suggests that these models can assist oncologists in making more informed clinical decisions.

## 4 Discussion

In our study, we developed two effective prediction models for LRR in BC. An important discovery and innovation of our study is the introduction of the NPI as a predictor of the LRR of BC, which has achieved ideal results. Our study has demonstrated that higher NPI scores are associated with higher LRR in BC in our real-world retrospective cohort. Additionally, we have preliminarily proven that NPI, as a predictor, performs better than TNM staging, which is an important prognostic indicator in BC.

Radiotherapy plays a crucial role in the comprehensive treatment of cancer. It is typically applied after surgical resection and often administered concurrently with chemotherapy and/or immunotherapy to achieve optimal tumor control (24). Among the advancements in radiotherapy technology, intensity-modulated radiation therapy (IMRT), a radiation therapy technology that modulates radiation intensity, has been widely used in cancer treatment. IMRT can preserve organs at risk and increase the radiation dose to the tumor, allowing for high-precision radiotherapy (25, 26). Additionally, hypofractionated radiotherapy based on IMRT and volumetric-modulated arc therapy technology has greatly improved the precision of BC treatment (27, 28). However, it is important to acknowledge that cancer recurrence after radiotherapy remains a significant form of treatment failure in most cases (29).

The recurrence probability for patients with BC after radiotherapy is approximately 20% - 30%, with the specific value depending on the patient’s condition, treatment effectiveness, and individual constitution (30, 31). One major reason for cancer recurrence after radiotherapy is the development of primary or secondary tolerance to radiation by tumor cells, known as radiation resistance (32). Many studies have reported the significant impact of LRR on cancer prognosis (33, 34). Patients with radiation-resistant tumors generally have worse survival rates, higher recurrence rates, and even higher rates of distant metastasis compared to radiosensitive patients (35, 36). Tumor cells with radioresistance can evade cell death after radiotherapy, leading to treatment failure. This issue is compounded by the accelerated repopulation of tumors, which primarily involves a group of residual radioresistant cancer cells, significantly reducing the sensitivity of recurrent tumors to treatment and resulting in poor clinical outcomes (37). Consequently, it is essential to understand the mechanisms whereby anti-radiation cells contribute to tumor repopulation to improve the prognosis of patients with cancer (38).

The heterogeneity of tumors makes it very difficult to predict the LRR of cancer (39). While prediction models based on genetic alterations or biomarkers have played an important role in predicting LRR in BC, it is undeniable that these prediction models often require patients to undergo gene expression testing, which greatly limits their clinical application (38, 40). Another difficulty in predicting LRR of BC is that radiotherapy in BC is primarily administered postoperatively. In this scenario, the patient’s tumor is usually completely resected, making the prediction of LRR quite challenging. Considering the ongoing controversy surrounding LRR prediction in BC, our study addresses this gap by constructing prediction models for locoregional recurrence of BC using common clinicopathological indicators. The results of our study demonstrate the effectiveness of these models in predicting LRR.

Many clinicopathological factors can significantly impact LRR (35). It is generally believed that as tumor invasion increases, tumor size increases, the proportion of hypoxic cells in tumor tissue increases, and the probability of tumor resistance to radiotherapy increases, which may be a leading factor in most cases of locoregional recurrence (33). In our study, we found a significant correlation between LRR of BC and pre-treatment T stage, specifically tumor size. This correlation has been confirmed in numerous radiobiological experiments (41). In addition, we found a significant correlation between lymph node stage and LRR. Generally, as cancer depth of invasion (T stage) increases, the rate of lymph node metastasis also increases (42). Many studies have shown that tumor invasion depth and differentiation degree are independent factors affecting cancer lymph node metastasis (43). Consequently, our study further highlighted TNM stage as an important clinical factor affecting LRR (**Table 2**). By incorporating differentiation grades and the presence of perineural invasion and vascular invasion as indicators of radioresistance, we conclude that tumor invasion ability is a major factor influencing LRR.

In the present study, we found that age, BMI, and estrogen receptor status differed among different LRR groups. Specifically, there was a positive correlation between patients’ age, BMI, and LRR, although the specific mechanism remains unclear. We speculate that hormone levels may play a significant role, as hormone status is associated with BC prognosis. Furthermore, age, BMI, and estrogen receptor status are highly correlated with female hormone levels and BC pathogenesis (44, 45). In addition, in most cases, the molecular subtype was found to be a significant predictor of BC. In our study, the proportion of triple-negative BC showed an increasing trend in the recurrence group, although this trend was not statistically significant (*P* = 0.062). This may be due to insufficient sample size. Specifically, the proportion of patients with BC and negative expression of estrogen and progesterone was significantly higher in the recurrence group, indicating that hormone status is an important factor affecting recurrence. However, in our univariate analysis, we found that hormone status did not affect locoregional recurrence of BC after radiotherapy. Additionally, whether patients received endocrine therapy or not did not affect radiotherapy recurrence. This suggests that the mechanism of the influence of hormone expression on radiotherapy recurrence requires further research. Furthermore, only 14.29% of patients in our study had triple-negative BC and did not receive any endocrine therapy. This limited sample size may have prevented us from observing the influence of triple-negative BC on radiotherapy recurrence. Consequently, due to the negative results regarding the impact of endocrine therapy on recurrence in our univariate analysis, we did not include it as a variable in our multivariate model.

In contrast to the TNM staging system, the NPI is calculated based on tumor size, number of lymph node metastases, and degree of tumor differentiation. Previous studies have shown that NPI scores are associated with poor prognosis in BC (12, 13). In our study, we compared the distribution of NPI scores in patients with BC and different recurrent statuses as well as explored the correlation between NPI scores and important pathological features. We found that NPI scores were highly correlated with tumor invasion and lymph node metastasis in BC. Furthermore, the recurrent BC population had a higher NPI score, which led us to consider building a prediction model based on NPI. During the modeling process, we encountered significant collinearity between NPI scores and TNM stages, and their predictive effects would be compromised if considered together. Therefore, we built separate prediction models and compared their efficiency. Through univariate logistic regression analysis, we successfully identified six important variables that influence prognosis, including age, BMI, TNM stage, NPI, vascular invasion, and perineural invasion. After conducting multivariate logistic regression analysis, we found that vascular invasion was not an independent predictor of radioresistance and, therefore, excluded it from the models. Consequently, we constructed two significant prediction models: TNM-stage- and NPI-based models. The participating variables in the TNM-stage-based model were age, BMI, TNM stage, and perineural invasion. In contrast, the NPI-based model included age, BMI, perineural invasion, and NPI. While the composition of the two prediction models was highly consistent, the NPI-based model demonstrated better prediction accuracy and robustness than the TNM-stage-based model.

In recent years, the application of nomogram model in the field of cancer has gradually increased (46, 47). It incorporates various clinicopathological or genetic factors that affect the onset, prognosis or recurrence of patients into the prediction model and visualizes them, quantifies the risk ratio into specific scores, and obtains the risk probability to predict disease recurrence, metastasis and prognosis through simple calculation, providing a convenient and beneficial tool for clinicians and researchers (48, 49). The results of model validation in our study suggest that NPI-based model has good consistency and discrimination in predicting the status of radioresistance. In addition, the decision curve analysis method showed that the use of nomogram assessment could bring higher clinical benefits under a certain risk threshold.

Our findings further broaden the application scope of the NPI in BC and improve its clinical value. However, there are some limitations to our study that should be mentioned. First, the retrospective nature of this study introduces potential selection bias. Additionally, the sample size was insufficient to meet the criterion of ≥10 patients per risk factor, although we attempted to include important positive cases within the study period. Second, as the data for this study were from a single center, they may not fully represent the broader population. Furthermore, our prediction model only underwent internal validation; therefore, the selection bias present in the training cohort may also exist in the validation cohort. Further external validation in a multicenter setting is needed to determine if this nomogram can be widely used in other populations. Lastly, Gunda et al. conducted a similar study on the locoregional recurrence risk predicted by NPI, which showed that the NPI can effectively predict the locoregional recurrence of BC. Furthermore, two published studies have confirmed that the NPI can effectively predict the distant metastasis of BC (13, 50). Notably, our study focused solely on locoregional recurrence and did not address distant metastasis. Therefore, it may be necessary to verify the predictive value of the NPI for distant metastasis in our cohort in the future.

## 5 Conclusion

In summary, a higher NPI score is an independent risk factor for predicting locoregional recurrence in BC. Two nomogram prediction models related to radioresistance were constructed in this study. The nomogram prediction model based on the NPI has undergone internal validation and has been found to have good discrimination and calibration. It has the potential to provide clinical benefits and merits widespread use and application.

## Data Availability

The original contributions presented in the study are included in the article/**Supplementary Material**. Further inquiries can be directed to the corresponding author.
